# Fasting plasma C‐peptide correlates with body mass index, hsCRP, apolipoprotein B, and other atherogenic lipids in healthy individuals

**DOI:** 10.14814/phy2.70282

**Published:** 2025-03-25

**Authors:** Per A. Whiss, Evangelia Baldimtsi, Jeanette Wahlberg

**Affiliations:** ^1^ Department of Biomedical and Clinical Sciences, Division of Clinical Chemistry and Pharmacology Linköping University Linköping Sweden; ^2^ Department of Acute Internal Medicine and Geriatrics in Linköping, and Department of Health, Medicine and Caring Sciences Linköping University Linköping Sweden; ^3^ Faculty of Medical Sciences Örebro University Örebro Sweden

**Keywords:** apolipoprotein B, body mass index, C‐peptide, healthy volunteers, lipoproteins

## Abstract

C‐peptide has a complex role in human physiology, but its effects are not fully understood. Studies have shown a protective impact against diabetic complications, but also that C‐peptide levels associate with cardiovascular events. Among the many applications to assess cardiovascular risk, circulating lipids are widely used, and one of the strongest biomarkers is apolipoprotein B. The aim of this investigation was to study the association of C‐peptide with markers of metabolic, inflammatory, or cardiovascular alterations in a limited group of healthy individuals. Body mass index (BMI), lipids, and other plasma markers were studied in 28 consecutive healthy individuals within the age of 30–50 years. The results showed significant positive correlations between C‐peptide and BMI (*r* = 0.498; *p* = 0.007); hsCRP (*r* = 0.530; *p* = 0.004); triglycerides (*r* = 0.530; *p* = 0.005); cholesterol (*r* = 0.507; *p* = 0.006), LDL‐cholesterol (*r* = 0.550; *p* = 0.002), LDL/HDL ratio (*r* = 0.460; *p* = 0.014); apoB (*r* = 0.622; *p* < 0.001), apoB/apoA1 ratio (*r* = 0.563; *p* = 0.002); and non‐HDL cholesterol (*r* = 0.566; *p* = 0.002). According to BMI values, 16 of the 28 individuals were overweight (BMI >25.0 kg/m^2^). If overweight individuals were excluded, C‐peptide did only correlate with apoB (*r* = 0.636; *p* = 0.026). To conclude, C‐peptide within normal levels associate with BMI and atherogenic lipids in healthy individuals, and apoB associate with C‐peptide even at normal weight. These results suggest that C‐peptide can be an early additional cardiovascular risk marker.

## 1 INTRODUCTION

C‐peptide is a bioactive endogenous peptide cleaved from proinsulin during the biosynthesis of insulin. Thereby, both insulin and C‐peptide are released to the circulation in equimolar amounts (Vejrazkova et al., 2020; Chen et al., 2023 for reviews). After release, C‐peptide exhibits a nearly tenfold half‐life compared to insulin. Studies have shown that C‐peptide has numerous effects on various cells and tissues, including antioxidant, antiapoptotic, and inflammatory properties (Vejrazkova et al., 2020; Chen et al., 2023; Dakroub et al., 2024 for reviews).

C‐peptide levels are reported to relate to BMI and age in type 1 diabetes (Kurpiewska et al., 2023; Sosenko et al., 2018), but it appears that C‐peptide has a complex and unclear relation to diabetic complications (Maddaloni et al., 2022). For example, levels of C‐peptide were reported to be positively associated with cardiovascular disease but inversely associated with diabetic retinopathy progression in type 2 diabetes (Wang et al., 2020). Both deficiencies and overproduction are probably involved in the complications (Chen et al., 2023), and therapeutic possibilities have also been suggested for C‐peptide in diabetes (Wahren & Larsson, 2015).

Several earlier studies have reported that increased blood levels of C‐peptide associate with cardiovascular disorders even in adults without diabetes (de Cabrera León et al., 2015; Min & Min, 2013; Patel et al., 2012), suggesting that C‐peptide can be an early marker of cardiovascular events in the general population. Plasma or serum C‐peptide levels have also been reported to associate with fat distribution (Donatelli et al., 1991; Li et al., 2014) and stroke in nondiabetic subjects (Li et al., 2014). Furthermore, a recent meta‐analysis of cross‐sectional studies showed a significant association between C‐peptide and an increased risk of cardiovascular events (Jahromi et al., 2023). Since C‐peptide has several effects on various cells and tissues, the cause of how C‐peptide increases cardiovascular risk is probably multifactorial, but one plausible and prominent part is through lipid metabolism.

There are many different applications to assess circulating lipoproteins for risk management. It appears clear that the cumulative burden of circulating low‐density lipoprotein cholesterol (LDL‐c) drives the development of atherosclerosis and the following cardiovascular events (Borén et al., 2020). Several studies have found that the ratio of LDL‐c to high‐density lipoprotein cholesterol (HDL‐c) is a better predictor for cardiovascular risk than LDL‐c or HDL‐c alone (Fernandez & Webb, 2008; Sun et al., 2022).

The major protein component of LDL‐c is apolipoprotein B (apoB). Measurement of apoB can be used to estimate the number of LDL‐c particles, but apoB also includes chylomicron remnants, intermediate‐density lipoproteins (IDL), very low‐density lipoproteins (VLDL), and lipoprotein(a) [Lp(a)] (Langlois et al., 2018). Elevated apoB and the apoB/apoA1 ratio are reported to strongly associate with major adverse cardiovascular events in both women and men (Walldius et al., 2021). Some of the more recent additions to the standard lipid panels in clinical laboratories is the report of non‐HDL‐c (Langlois et al., 2018) and remnant cholesterol (Stürzebecher et al., 2023), but apoB is increasingly recognized as the strongest risk markers for cardiovascular disease (Sniderman et al., 2024).

Fasting levels of serum C‐peptide have been shown to negatively associate with HDL‐c and increased cardiovascular risk (Li et al., 2015), and with “atherogenic index” (cholesterol‐HDLc/HDL‐c) in healthy individuals (Kron et al., 2021). Furthermore, serum levels of both C‐peptide and the marker of low‐grade inflammation, hsCRP, have recently been reported to associate with both all‐cause mortality and cardiovascular events in early type 2 diabetes (Gedebjerg et al., 2023).

The purpose of the present study was to investigate the association of fasting C‐peptide with circulating levels of lipids and other markers of metabolic, inflammatory, cardiovascular, or renal alterations in a small group of healthy individuals with a limited age range.

## 2 MATERIALS AND METHODS

### 2.1 Subjects and blood sampling

The study population consisted of 28 subjectively healthy volunteers included on a consecutive basis at the Department of Endocrinology in Linköping, Sweden. The study subjects were informed about the purpose of the study and gave written consent to participate. The protocol was approved by the regional ethical committee. Inclusion criteria were age between 30 and 50 years, and the aim was even sex distribution. Exclusion criteria were pharmacological treatment, the use of drugs, and alcohol abuse. None of the participants stated that they were smokers. Blood and urine were sampled after 10 h of fasting.

### 2.2 Analytical measurements

Urine samples included an analysis of U‐albumin/creatinine ratio. Blood samples were drawn into lithium heparin tubes and centrifuged at 1500*g* for 15 min. All analyses were measured using standard assays at the Laboratory of Clinical Chemistry at the University Hospital in Linköping, Sweden. Plasma concentrations of C‐peptide were analyzed using cobas e602. C‐reactive protein (CRP), cholesterol, triglycerides, LDL‐c, HDL‐c, apolipoprotein‐A1 (apo‐A1) and apolipoprotein‐B (apo‐B) were analyzed utilizing the clinical chemistry analyzer Advia 1650 from Roche. LDL cholesterol was calculated using the Friedewald equation. No individual had triglycerides >4 mmol/L. Remnant cholesterol was calculated as total cholesterol minus LDL cholesterol minus HDL cholesterol, and non‐HDL‐c was calculated as remnant cholesterol + LDL‐c (Wadström et al., 2023).

### 2.3 Statistics

GraphPad Prism® was used for statistical analysis (version 10.0.2; GraphPad Software Inc., San Diego, Ca). Normality was evaluated in every quantitative variable by means of the Shapiro–Wilk test. Plasma C‐peptide did not pass the normality test (alpha = 0.05) and the relationship between factors was investigated with nonparametric Spearman correlation and a two‐tailed 95% confidence interval. A *p* value of <0.05 was judged as statistically significant.

## 3 RESULTS

Most individuals exhibited values of the various analytes within the reference intervals (included in Table 1) with the following exceptions: Two individuals had fasting C‐peptide levels above the reference (1.4 and 1.6 nmol/L); one individual had IL‐6 above the reference (12 pg/mL); and two individuals had elevated fibrinogen (4.4 and 5.0 g/L). Eight individuals had increased systolic blood pressure within the range 122–139 mmHg, and two individuals had higher diastolic blood pressure (both had 91 mmHg). All individuals had lipid and lipoprotein levels within the reference intervals, except for one woman with remnant cholesterol above the reference (1.2 mmol/L). Median, distribution, and reference intervals are shown in Table 1.

**TABLE 1:** Healthy volunteers characteristics.

	Median (interquartile range)	Reference interval*
*N*	28	–
Females *n* (%)	15 (54%)	
Age (years)	38.0 (9.2)	–
Body mass index (kg/m^2^)	25.3 (6.0)	18.5–24.9**
Waist‐hip ratio	0.85 (0.08)	Female <0.85***
		Male <0.90***
fP‐glucose (mmol/L)	5.4 (0.6)	4.2–6.0
P‐HbA1c (mmol/mol)	33.0 (4.0)	27–42
fP‐C‐peptide (nmol/L)	0.65 (0.22)	0.12–1.20
eGFR MDRD (mL/min/1.73 m2)	82.0 (15.0)	>60
Ratio U‐albumin/U‐creatinine (mg/mmol)	0.28 (0.16)	<5
P‐hsCRP (mg/L)	0.70 (1.42)	<10
P‐fibrinogen (g/L)	2.80 (0.65)	2–4
P‐interleukin‐6 (ng/L)	1.00 (1.02)	<7
fP‐triglycerides (mmol/L)	0.83 (0.57)	0.45–2.60
P‐cholesterol (mmol/L)	4.55 (0.92)	3.3–6.9
P‐LDL‐c (mmol/L)	2.55 (0.95)	1.4–4.7
P‐HDL‐c (mmol/L)	1.40 (0.40)	Female 1.0–2.7
		Male 0.8–2.1
Ratio LDL‐c/HDL‐c	1.64 (0.98)	0, 4–6, 6
P‐apolipoprotein A1 (g/L)	1.37 (0.26)	Female 1.2–2.1
		Male 1.0–1.8
P‐apolipoprotein B (g/L)	0.84 (0.29)	0.6–2.0
Ratio apoB/apoA1	0.57 (0.20)	Female <0.8
		Male <0.9
Ratio apoB/LDL‐c	0.33 (0.03)	Data not available
Ratio apoA1/HDL‐c	0.99 (0.19)	Data not available
P‐non‐HDL‐c (mmol/L)	3.05 (1.10)	Female 1.9–5.1****
		Male 2.0–6.1****
P‐cholesterol remnants (mmol/L)	0.4 (0.3)	Female 0.19–0.94****
		Male 0.22–1.83****
Systolic blood pressure (mmHg)	111.5 (22.8)	<120
Diastolic blood pressure (mmHg)	73.0 (9.0)	<80

* According to Laboratory Medicine, Region Östergötland, Sweden, unless otherwise stated.

** According to the Public Health Agency of Sweden and the WHO.

*** According to “Waist circumference and waist‐hip ratio”. Report of a WHO Expert Consultation Geneva; 8–11 December 2008.

**** According to Ridefelt et al., 2019.

Sex‐dependent differences were evident for plasma levels of fibrinogen, triglycerides, LDL‐c, LDL‐c/HDL‐c ratio, apoB/apoA1‐ratio, non‐HDL‐c, and cholesterol remnants, as well as waist– hip ratio and systolic blood pressure (Table 2). The levels of C‐peptide showed no sex‐dependent difference, median (interquartile range) was 0.63 (0.24) nmol/L for females, and 0.67 (0.20) for males.

**TABLE 2:** Healthy volunteers characteristics with sex‐dependent differences.

	Median (interquartile range)		*p* Value	Reference interval*
	Female	Male		
*N*	15	13	–	–
Waist‐hip ratio	0.84 (0.05)	0.89 (0.09)	0.033	Female <0.85**
				Male <0.90**
P‐fibrinogen (g/L)	3.00 (0.70)	2.50 (0.50)	0.049	2–4
fP‐triglycerides (mmol/L)	0.73 (0.33)	1.10 (0.71)	0.013	0.45–2.60
P‐LDL‐c (mmol/L)	2.10 (0.70)	2.80 (0.985)	0.014	1.4–4.7
Ratio LDL‐c/HDL‐c	1.43 (0.76)	2.15 (1.19)	0.018	0,4‐6,6
Ratio apoB/apoA1	0.53 (0.24)	0.64 (0.20)	0.031	Female <0.8
				Male <0.9
P‐non‐HDL‐c (mmol/L)	2.60 (0.90)	3.10 (1.11)	0.031	Female 1.9–5.1***
				Male 2.0–6.1***
P‐cholesterol remnants (mmol/L)	0.3 (0.1)	0.5 (0.4)	0.041	Female 0.19–0.94***
				Male 0.22–1.83***
Systolic blood pressure (mmHg)	104.0 (16.0)	119.0 (17.5)	0.009	<120

* According to Laboratory Medicine, Region Östergötland, Sweden, unless otherwise stated.

** According to “Waist circumference and waist‐hip ratio”. Report of a WHO Expert Consultation Geneva; 8–11 December 2008.

*** According to Ridefelt et al., 2019.

**FIGURE 1:**
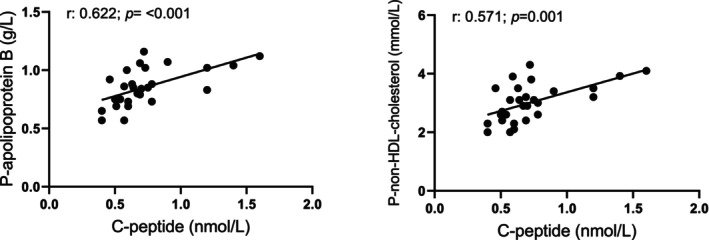
Correlation of fasting plasma C‐peptide with plasma apolipoprotein B and non‐HDL cholesterol in 28 subjectively healthy individuals.

Using nonparametric Spearman correlation, plasma C‐peptide levels showed a strong positive correlation with BMI as well as with fasting plasma levels of hsCRP, triglycerides, cholesterol, LDL‐c, LDL‐c/HDL‐c ratio, apoB, apoB/apoA1 ratio, and non‐HDL‐c (Table 3). Circulating apoB and non‐HDL‐c showed the strongest associations with C‐peptide (Figure 1). Age showed a positive correlation only for the levels of P‐fibrinogen (*r* = 0.467, *p* = 0.014).

**TABLE 3:** Relationship between fasting plasma C‐peptide concentration and various factors and tests in healthy individuals (*n* = 28).

	*r* with C‐peptide	*p*‐Value
Age (years)	‐0.035	0.859
Body mass index (kg/m^2^)	0.498	0.007
Waist‐hip ratio	0.191	0.330
fP‐glucose (mmol/L)	0.215	0.271
P‐HbA1c (mmol/mol)	0.274	0.158
eGFR MDRD	−0.000	0.999
Ratio U‐albumin/U‐creatinine)	−0.093	0.666
P‐hsCRP (mg/L)	0.530	0.004
P‐fibrinogen (g/L)	0.323	0.100
P‐interleukin‐6 (pg/mL)	0.121	0.540
fP‐triglycerides (mmol/L)	0.530	0.005
P‐cholesterol (mmol/L)	0.507	0.006
P‐LDL‐c (mmol/L)	0.550	0.002
P‐HDL‐c (mmol/L)	−0.253	0.195
Ratio LDL‐c/HDL‐c	0.460	0.014
P‐apolipoprotein A1 (g/L)	−0.026	0.897
P‐apolipoprotein B (g/L)	0.622	<0.001
Ratio apoB/apoA1	0.563	0.002
Ratio apoB/LDL‐c	0.042	0.832
Ratio apoA1/HDL‐c	0.331	0.085
P‐non‐HDL‐c (mmol/L)	0.571	0.001
P‐cholesterol remnants (mmol/L)	0.363	0.058
Systolic blood pressure (mmHg)	0.176	0.371
Diastolic blood pressure (mmHg)	0.017	0.933

*Note*: Nonparametric Spearman correlation (*r*) was used to determine whether values between variables were significantly associated (underlined indicates *p < 0.05*).

**FIGURE 2:**
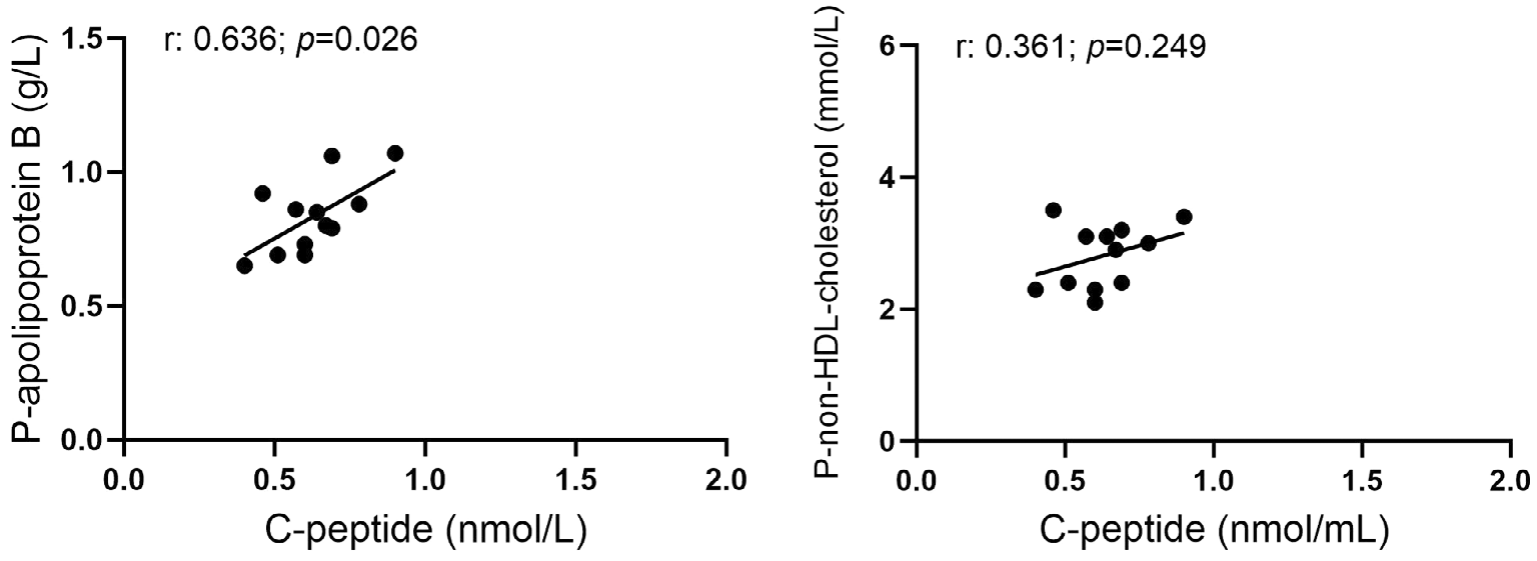
Correlation of fasting plasma C‐peptide with plasma apolipoprotein B and plasma non‐HDL cholesterol in 12 healthy volunteers with body mass index denoting normal weight (18.5–24.9).

**FIGURE 3:**
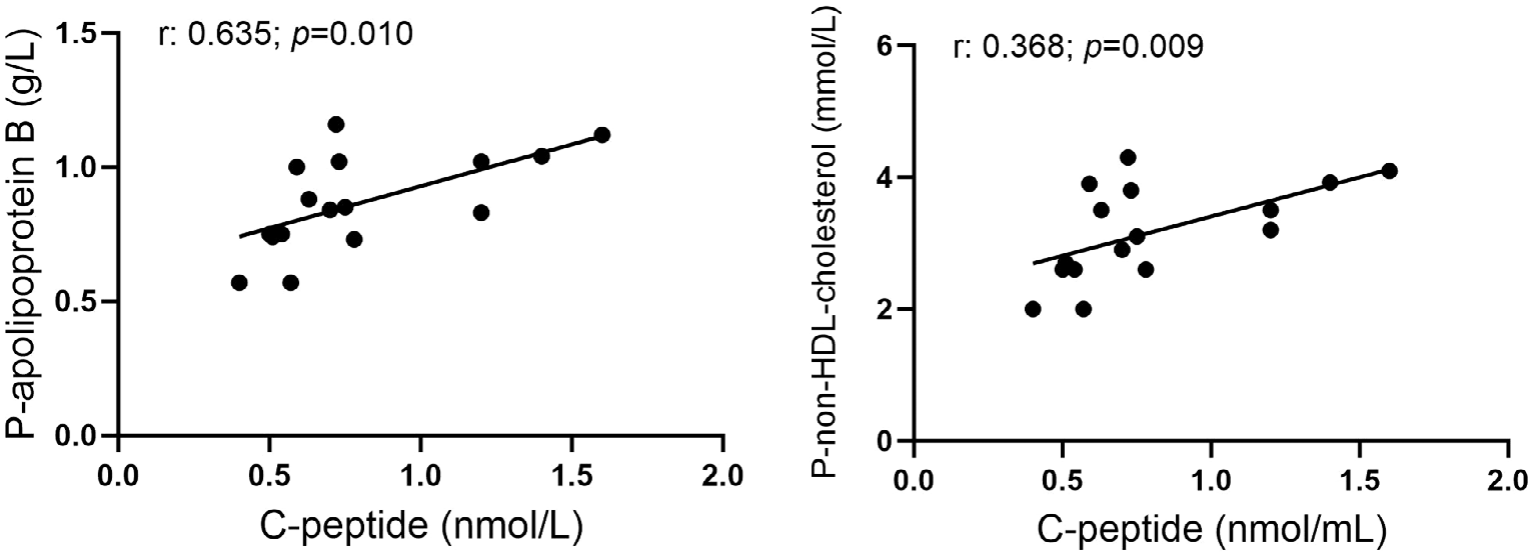
Correlation of fasting plasma C‐peptide with plasma apolipoprotein B and plasma non‐HDL cholesterol in 16 healthy volunteers with a body mass index denoting overweight (>25).

According to levels specified by the World Health Organization, 16 (9 women and 7 men) of the 28 participating volunteers that were consecutively included in this study exhibited BMI defining overweight (BMI > 25.0 kg/m^2^). Of these 16 overweight individuals, three women, and two men were classified as obese (BMI of 30.0 or higher). Twelve volunteers were classified as normal weight, and none as underweight. Upon exclusion of obese individuals, the significant correlations of C‐peptide with BMI, LDL‐c/HDL‐c ratio, triglycerides, and hsCRP were lost, but the correlation of C‐peptide with cholesterol, LDL‐c, apoB, apoA1, apoB/apoA1 ratio, and non‐HDL‐c remained (Data not shown). If overweight individuals were also excluded, C‐peptide correlated only with apoB (Figure 2). The correlation of C‐peptide with apoB and non‐HDL‐c in the subgroup with overweight is shown in Figure 3.

## 4 DISCUSSION

Circulating levels of fasting C‐peptide can be an important risk factor for cardiovascular disease or overall death. In the present study, C‐peptide levels in plasma showed a strong positive correlation with BMI, hsCRP, cholesterol, LDL‐c, LDL‐c/HDL‐c ratio, apoB, apoB/apoA1 ratio, non‐HDL‐c, and fasting triglycerides. The correlation between C‐peptide and cholesterol, LDL‐c, apoB, apoA1, apoB/apoA1 ratio, and non‐HDL‐c remained upon exclusion of obese individuals. However, upon exclusion of overweight individuals, the only correlation of C‐peptide that remained was with apoB.

Seventeen of the 28 participating unselected healthy individuals were classified as overweight according to values specified by the Public Health Agency of Sweden. It is estimated that around 50% of adults in Sweden are overweight or obese (Public Health Agency of Sweden, 2018), indicating that the small sample of individuals in the present study is quite representative. These numbers are also similar with more recent data from the European population (European Union, 2024).

In well‐controlled type 2 diabetic patients, serum C‐peptide levels have earlier been reported to significantly correlate with BMI (*r* = 0.21), HDL‐c (*r* = −0.22), non‐HDL‐c (*r* = 0.23), triglycerides (*r* = 0.39), apoB (*r* = 0.29), and systolic and diastolic blood pressure (Relimpio et al., 1997). Dyslipidemic individuals with higher apoB levels have earlier been reported to have higher levels of C‐peptide, as well as higher BMI (Vaverkova et al., 2009). Apart from the negative correlation of C‐peptide with HDL‐c in type 2 diabetics, the present study shows that these earlier reported associations also are present in healthy adults.

Several studies have indicated that the LDL‐c/HDL‐c ratio is a more robust predictor of cardiovascular disease than the individual levels of LDL‐c and HDL‐c (Sun et al., 2022). ApoB represents all atherogenic lipoprotein particles and includes triglyceride‐rich lipoproteins such as LDL, VLDL, IDL, and Lp(a) and their remnants (Langlois et al., 2018). Correspondingly, the ratio apoB/LDL‐c has been suggested to give an idea of the size distribution within the LDL‐c population, and a high ratio indicates an increased incidence of atherogenic small LDL‐c. Plasma concentration of apoB and the apoB/apoA1 ratio have been reported to strongly relate to increased risk of fatal myocardial infarction in both men and women (Walldius et al., 2021). In the same study, apoA1 was found to be protective. Furthermore, elevated LDLapoB/LDL‐c ratio have been reported to associate with increased cardiovascular mortality in patients referred for coronary angiography (Silbernagel et al., 2022), and levels of apoB are increasingly recognized as the strongest risk markers for cardiovascular disease (Sniderman et al., 2024).

The effect of C‐peptide on cardiovascular risk has been suggested to be bidirectional, that is, to play a beneficial role at low levels, and play a harmful role at a high level in nondiabetic adults and patients with newly diagnosed type 2 diabetes (Yan et al., 2022). High levels of C‐peptide depend on hyperinsulinemia, which in turn is associated with altered lipid metabolism, hypertension, and overweight. Several studies have reported that plasma apoB can predict type 2 diabetes, and plasma apoB has been reported to correlate with total insulin and C‐peptide in obese healthy individuals with a BMI > 27 kg/m^2^ (Bissonnette et al., 2015). The present study shows that the association of apoB with normal levels of C‐peptide is highly significant even in healthy individuals with normal weight, whereas the association of C‐peptide with all other analytes was dependent on overweight, suggesting that C‐peptide can be an additional early risk marker for cardiovascular disease. C‐peptide may therefore be an additional risk marker, especially for normal weight individuals, but prospective studies with larger sample sizes are required to confirm its applicability.

## AUTHOR CONTRIBUTIONS

EB and JW contributed to the study design. EB and PW analyzed the data. PW wrote the first draft of the manuscript. PW, EB, and JW revised the manuscript. All authors approved the final version of the manuscript.

## FUNDING INFORMATION

This study received no external funding. Open access funding was provided by Linköping University.

## CONFLICT OF INTEREST STATEMENT

The authors declare no conflicts of interest.

## ETHICS STATEMENT

The protocol received approval from the regional ethical committee in Linköping. Test results used in this study were pseudonymized.

## CONSENT

Informed consent to take part in the study was obtained from all participants.

## Data Availability

Data presented in the present study are available from the corresponding author upon reasonable request.
